# Investigating the Role of Genetic Polymorphisms in External Apical Root Resorption Among Orthodontic Patients: Implications for Treatment Outcomes—A Literature Review

**DOI:** 10.3390/reports8010014

**Published:** 2025-01-24

**Authors:** Christina Charisi, Vasileios Zisis, Konstantinos Poulopoulos, Stefanos Zisis, Athanasios Poulopoulos, Dieter Müßig

**Affiliations:** 1 Department of Orthodontics, School of Dentistry, Danube Private University, 3500 Krems, Austria; charisidentist@gmail.com (C.C.);; 2 Department of Oral Medicine and Pathology, School of Dentistry, Aristotle University of Thessaloniki, 54124 Thessaloniki, Greeceakpoul@dent.auth.gr (A.P.); 3 Department of Periodontology, School of Dentistry, Aachen University, 52074 Aachen, Germany

**Keywords:** root resorption, apical, external, orthodontically induced, orthodontic treatment, gene polymorphisms

## Abstract

**Background:** Among the various forms of root resorption, External Apical Root Resorption (EARR) has garnered particular attention due to its prevalence and potential complications associated with orthodontic interventions. **Methods:** An electronic search of literature was performed between September 2024 and December 2024 to identify all articles investigating the Role of Genetic Polymorphisms in External Apical Root Resorption Among Orthodontic Patients: Implications for Treatment Outcomes. The search was conducted using MEDLINE (National Library of Medicine)-PubMed with restrictions concerning the date of publication. In particular, we focused on the period 2014–2024 using the following keywords: gene polymorphisms AND orthodontic treatment AND apical root resorption OR external apical root resorption. This was followed by a manual search, and references were used to identify relevant articles. **Results:** The review showed that certain variations of the following genes may be positively associated with OIEARR: Osteopontin gene, P2RX7, IL-1β, IL-6, IL1RN, OPG, RANK, STAG2, RP1-30E17.2, SSP1, SFRP2, TNFSF11, TNFRSF11A, TNFRSF11B, VDR, CYP27B1, ACT3N, TSC2, WNT3A, LRP1, LRP6. Conversely, the IRAK1 gene has a protective function against the development of OIEARR. **Conclusions:** Despite these advancements, it is still not feasible to establish new guidelines and clinical protocols based on the existing research findings. The integration of genetic considerations into orthodontic practice has the potential to revolutionize treatment strategies, ensuring that they are not only effective but also respectful of each patient’s unique biological landscape.

## 1. Introduction and Clinical Significance

Apical root resorption (ARR) is a complex condition primarily associated with orthodontic treatment, where it emerges as one of the most undesirable side effects due to its impact on dental health and patient outcomes [1,2]. Defined as the permanent loss of cementum and dentine at the tooth’s root apex, ARR chiefly affects the anterior teeth, with the maxillary incisors being the most susceptible [1,2]. The prevalence of ARR during orthodontic treatment is significant, with reported cases ranging widely from 20% to 100% among patients [2]. This variability underscores the need for orthodontists to closely monitor and manage this condition to minimize potential damage. Despite its prevalence, the phenomenon remains poorly understood, particularly in the context of treatment with clear aligners, where the mechanisms and extent of ARR differ from traditional fixed appliances [2]. Studies have shown that while fixed appliances often result in a mean root length loss of 2.26 mm in maxillary incisors, treatment with clear aligners tends to result in significantly less severe resorption [2]. This discrepancy highlights the critical role of treatment modality in influencing the severity of ARR. Preventive measures and guidelines are crucial to reducing the incidence and severity of ARR, thus safeguarding patients’ quality of life and ensuring the success of orthodontic interventions [1].

One of the most common factors predisposing individuals to ARR is the persistent trauma caused by habits such as tongue thrusting during orthodontic treatment [1]. Furthermore, even before undergoing orthodontic procedures, individuals with anterior open bite typically present with shorter root lengths in their maxillary central incisors, suggesting an intrinsic vulnerability to ARR [1]. Given these interconnected factors, it is imperative for orthodontic protocols to incorporate preventive strategies aimed at mitigating the impact of adverse oral habits and anatomical predispositions to reduce the risk of ARR.

ARR can be categorized into different types based on its etiology and severity, and understanding these types is essential for tailoring orthodontic interventions. One type of ARR is related to the duration and type of orthodontic treatment, where prolonged treatment time and specific force directions can contribute significantly to the risk of resorption [3]. Furthermore, genetic factors also influence ARR, as certain gene polymorphisms have been associated with a predisposition to resorption, suggesting that genetic screening could be a valuable tool in predicting and managing this condition. Recognizing these types and their interconnections highlights the need for personalized treatment plans that consider both mechanical and genetic factors to mitigate the risk of severe ARR.

### 1.1. External Apical Root Resorption (EARR) in Orthodontic Patients

External apical root resorption (EARR) represents a significant challenge within orthodontic treatment, manifesting as the reduction in root structure primarily in the apical region, which can be detected using standard radiographs [4]. The process of detecting EARR is crucial, involving careful assessment of the extent of root resorption during orthodontic treatment. The detection not only aids in identifying the severity of EARR but also in understanding its clinical significance, particularly because it can affect the long-term prognosis of the treatment and stability of the tooth structure.

### 1.2. Evaluating the Efficacy of Radiographic Techniques in Diagnosing External Apical Root Resorption

In the evaluation of radiographic techniques for diagnosing external apical root resorption, a critical aspect is the comparative analysis of conventional and digital methods, each having its unique strengths and weaknesses. Conventional radiographs, while routinely used in clinical practice, are known to produce false-negative results in a significant percentage of cases (15.3%), which can impede timely diagnosis and treatment planning [5]. Digital radiographic methods, on the other hand, aim to enhance the detection of simulated external root resorption cavities, particularly in scenarios where small cavity sizes could potentially affect detectability [6]. However, both conventional and digital radiographs encounter limitations, especially in accurately identifying small defects on the buccal or lingual surfaces, as they may not offer sufficient sensitivity for such nuanced diagnosis [5]. In contrast, cone-beam computed tomography (CBCT) has been identified as a superior diagnostic tool, particularly in cases of suspected severe EARR, providing a more comprehensive assessment that is crucial for informed decision-making and effective treatment planning [7]. Despite the higher sensitivity of CBCT in detecting smaller root resorption cavities, some studies have noted that there are no statistically significant differences between imaging methods in the correct detection of external apical root resorption across various cavity sizes [8]. Thus, while CBCT offers enhanced diagnostic capabilities, the choice of radiographic method must consider the specific clinical context and the trade-offs between sensitivity and practicality in routine use.

### 1.3. The Relationship Between Genetic Polymorphisms and EARR

Genetic polymorphisms have emerged as key contributors to the risk of EARR during orthodontic treatment, emphasizing the complexity of genetic influences in dental health [9]. Among various genes studied, IL-1RN polymorphisms have been notably associated with EARR risk, with the TT genotype of the IL-1RN SNP rs419598 showing a strong positive correlation with the condition [5]. This correlation is thought to be due to the high expression of IL-1ra, which might alter bone resorption by blocking IL-1 from stimulating osteoclasts, thus increasing susceptibility to root resorption [4]. Despite these findings, the current body of research remains insufficient, with significant gaps that need to be addressed to fully understand the genetic underpinnings of EARR [9]. This necessitates further studies, particularly those focused on human genetic factors, to enable orthodontists to personalize treatments and accurately identify at-risk patients early on [4,9]. As such, enhancing our understanding of the molecular aspects and genetic variations linked to EARR could lead to improved diagnostic and therapeutic strategies in orthodontic care [9].

The genetic predisposition to orthodontically induced external apical root resorption (OIEARR) has been extensively studied, with significant findings highlighting the role of various interleukin-1 (IL-1) genes. Notably, the IL-1α gene, particularly its TT genotype, has been identified as a major contributor to EARR among German Caucasians, suggesting a genetic susceptibility linked to inflammatory responses during orthodontic treatment [4]. This association is further supported by the presence of the SNP rs1800587, which correlates with elevated IL-1 protein levels in the gingival crevicular fluid, indicating an enhanced inflammatory environment that could exacerbate root resorption [4]. Furthermore, multiple studies have consistently reported the involvement of the IL1 gene family, with the IL-1B gene emerging as another significant player. The C3954T polymorphism of IL-1B is particularly noteworthy, as it boosts IL-1B production, thereby intensifying catabolic bone remodeling in the periodontal ligament, which may contribute to the progression of EARR [4]. These findings underscore the importance of understanding genetic influences on EARR, suggesting that genotypic screening could potentially inform personalized orthodontic treatment plans to mitigate this adverse effect.

Research indicates that genetic factors significantly contribute to EARR, as evidenced by studies that have identified a correlation between interleukin gene polymorphisms, particularly in IL-1B, and the severity of EARR [10]. The IL-1B gene polymorphisms are among the most studied in relation to EARR, and while some studies have demonstrated a positive correlation, others have reported conflicting results, highlighting the complexity of genetic influences on EARR [10]. Additionally, the P2RX7 gene, which encodes a purinergic receptor involved in osteoblast and osteoclast activity, has been implicated in EARR development. This gene not only promotes osteoblast activity but also triggers apoptosis in osteoclasts, suggesting its role in bone remodeling processes that may influence the severity of EARR [10]. Despite these findings, the genetic landscape of EARR is complex, with studies suggesting that mutations in the IL-1B gene account for only a fraction of the phenotypic variation observed, indicating the involvement of other genetic factors [10]. This necessitates further research to clarify the role of additional genetic polymorphisms and understand the interplay between genetic predisposition and environmental factors such as orthodontic treatment in the development of EARR [10]. Understanding these genetic underpinnings could lead to personalized treatment strategies, minimizing the risk of EARR in susceptible individuals.

Our study aims to collect and review the original research articles that investigate genetic polymorphisms regarding their role in OIEARR. There are certain gaps in the existing literature: the first gap refers to the lack of an up-to-date review of all the existing information whereas the second gap refers to the need for new guidelines and clinical protocols based on this information. Therefore, the current study is going to tackle the first gap.

## 2. Material and Method

An electronic search of the literature was performed between September 2024 and December 2024 to identify all articles investigating the Role of Genetic Polymorphisms in External Apical Root Resorption Among Orthodontic Patients: Implications for Treatment Outcomes. The search was conducted using MEDLINE (National Library of Medicine)-PubMed with restrictions concerning the date of publication. In particular, we focused on the period 2014–2024 using the following keywords: gene polymorphisms AND orthodontic treatment AND apical root resorption OR external apical root resorption. This was followed by a manual search, and references were used to identify relevant articles. The articles identified from the electronic and manual search were screened to eliminate those that failed to meet the respective inclusion and exclusion criteria as listed below.

### Inclusion and Exclusion Criteria

The electronic search is based on the following criteria:

1. Language: articles written in English;
2. Time frame: studies published between 2014–2024;
3. Type of study: original research articles;
4. Study sample: at least 30 cases (*n* ≥ 30).

## 3. Results

In total, 430 articles were identified through the keywords. After the implementation of the time frame 2014–2024, 274 articles remained. Subsequently, by reading the titles and abstracts, and therefore by excluding the non-original research articles, 16 articles remained (Table 1).

Iglesias Linares et al. [11] showed that certain variations of the osteopontin gene (rs9138 and rs11730582) may determine a statistically significant genetic predisposition to develop OIEARR.

Sharab et al. [12] concluded that a lengthy treatment parallel to specific genotype for P2RX7 (rs208294) were statistically significantly associated with OIEARR.

Guo et al. [13] found no statistically significant association between gender, extent of tooth movement, and IL-1RN (rs419598) with OIEARR. On the other hand, IL-6 (rs1800796) was associated with OIEARR.

Pereira et al. [14], in contrast to Guo, found statistically significant correlations between OIEARR and gender, length of treatment, premolar extractions, and Hyrax appliance. Pereira’s most noteworthy finding refers to the protective function of the IRAK1 gene.

Iglesias Linares et al. [15] proved that clinical case complexity and the extent of incisor apical displacement in the sagittal plane are statistically significantly associated with OIEARR. No association was found with the type of orthodontic appliance used (fixed or removable). Furthermore, homozygosity of the IL1RN (rs419598) was correlated to OIEARR.

Borilova Linhartova et al. [16], in contrast to Iglesias Linares, showed an association between lengthy orthodontic treatment through fixed appliance and OIEARR. In addition, P2RX7 (rs208294 and rs1718119) increased the possibility of OIEARR. A significant limitation of this study is the inclusion of exclusively Czech children.

Borges de Castilhos et al. [17] discovered that the initial root length and the age were associated with OIEARR. No association was found between RANKL and OIEARR. RANK (12455775) and OPG (rs3102724, rs2875845, rs1032128, and rs3102728) were associated with OIEARR. However, after multivariate analysis, only the long roots of upper central incisors, rapid maxillary expansion, and OPG (rs3102724) were still associated with OIEARR.

Iber-Diaz et al. [18] revealed that two X chromosome genes, STAG 2 (rs151184635) and RP1-30E17.2 (rs55839915), are associated with OIEARR, specifically in males. On the other hand, SSP1 (rs11730582), P2RX7 (rs1718119), and TNFRSF11A (rs8086340) were marginally associated with OIEARR, exclusively in females. Therefore, a gender predilection was established.

Ciurla et al. [19,20] concluded that OIEARR may originate from IL-1β (which may increase the probability of OIEARR up to four times) as well as P2RX7 (rs208294) and IL1RN (rs419598).

Silva et al. [21] deduced that the most crucial cofounding factor was the duration of the treatment followed by the gender and the implementation of premolar extraction. Regarding osteoprotegerin (OPG), the receptor activator of nuclear factor κ B (RANK), and the IL1 receptor antagonist (IL1RN), their effect could range from protective to harmful, depending on the duration of treatment and the age of the patient.

Yun-Ju Lee et al. [22] did not result in any statistically significant association among clinical confounding factors and OIEARR. SPP1 (rs9138) and SFRP2 (rs3810765) may be associated with OIEARR, although marginally, since the (after Bonferroni correction) multiple testing did not result in any significant findings. In general, both genes associated with inflammation and the Wnt signaling may provoke OIEARR.

Maranon Vasquez et al. [23] found that AA VDR (rs2228570) had a 21% higher probability of inducing OIEARR, whereas heterozygous VDR (rs2228570) manifested a 12% lower probability of inducing OIEARR than that of homozygotes. A specific haplotype VDR (rs1544410-rs7975232-rs731236) manifested a 16% lower probability. Homozygotes AA CYP27B1 (rs4646536) manifested a higher probability as well. Multiple testing erased any statistically significant correlations; nevertheless, possible correlations may be extracted as a result of this study.

Liu Jing et al. [24] resulted in 11 SNPs regarding the genes TNFSF11, TNFRSF11B, WNT3A, SFRP2, LRP6, P2RX7, and LRP1 that may be associated with OIEARR in the Korean population. The obvious limitation of this study, as with the previous one from Borilova Linhartova, is that it includes only a specific ethnic group, Koreans, in this case.

Jyoti Chauhan et al. [25] assessed the presence of IL-1b (rs1143634) and IL-1a (rs1800587) in patients exhibiting OIEARR but resulted in no correlation.

John M Burnheimer et al. [26] deduced that Class III malocclusion and lengthy orthodontic treatment may affect the probability of OIEARR. ACT3N (rs678397) and TSC2 (rs1051771) were statistically significantly associated with OIEARR.

## 4. Discussion

ERR constitutes a detrimental complication associated with orthodontic treatment, characterized by the loss of tooth root structure, which can compromise long-term dental health and stability. Recent advancements in genetic research have illuminated the potential role of single nucleotide polymorphisms (SNPs) in influencing an individual’s susceptibility to ERR, particularly in orthodontic patients. Among the myriad of genetic factors, the osteopontin gene has emerged as a significant player, with specific SNPs, namely rs9138 and rs11730582, garnering attention for their potential association with ERR [11]. These SNPs may modulate the expression of osteopontin, a glycoprotein involved in bone remodeling and mineralization, thereby affecting the biological response to orthodontic forces. Understanding the mechanisms by which these SNPs operate is crucial, as they may interact with both genetic predispositions and environmental factors, exacerbating the risk of root resorption during orthodontic treatment.

The influence of SNPs rs9138 and rs11730582 on susceptibility to EARR may be contingent upon ethnic background, as evidenced by contrasting findings across populations. The investigation conducted by Linhartova et al. in the Czech population found no significant association between these specific SNPs of the osteopontin (OPN) gene and EARR, suggesting that these genetic variations might not contribute to EARR susceptibility in this ethnic group [4]. Conversely, research by Iglesias-Linares et al. indicates that in Caucasian populations, the inheritance of particular alleles of the OPN gene, notably SNPs rs9138 and rs11730582, could potentially modulate genetic susceptibility to EARR [4]. These disparate findings underscore the complexity of genetic influences on dental pathologies and highlight the importance of considering ethnic diversity when assessing genetic risk factors. Understanding the interaction between genetic predispositions and ethnicity is crucial for developing more precise diagnostic and therapeutic strategies tailored to individual genetic profiles.

The mechanism by which SNPs affect orthodontic treatment outcomes is multifaceted, involving complex genetic and molecular interactions. Among these, the SNP rs9138, a 3ʹ-UTR variant, has been identified as a key regulator influencing the degradation of SPP1 through miRNA mediation, subsequently affecting protein levels [27]. This SNP’s influence extends beyond molecular degradation to have practical implications in orthodontics, notably its association with EARR [27]. This connection highlights the SNP’s potential role in modifying bone biology, thereby impacting orthodontic tooth movement [27]. The involvement of SPP1 in encoding osteopontin (OPN), a protein critical for bone resorption processes during orthodontic treatment, suggests that genetic variations in SPP1 can alter the dynamics of alveolar bone remodeling [27]. This remodeling occurs at both the compression and tension sides of the alveolar bone, responding to orthodontic stress and ultimately influencing the rate of tooth movement [27].

The IL1RN gene encodes the IL1 receptor antagonist (IL1Ra), which is crucial in inhibiting the activation of osteoclasts by IL-1, a process that, if unchecked, can lead to bone resorption and ultimately root resorption [20]. This genetic modulation is particularly evident in the TT genotype of the IL1RN SNP rs419598, which has been shown to have a strong positive correlation with EARR, suggesting that individuals with this genotype may be at an increased risk of developing root resorption during orthodontic treatment [20].

Furthermore, the investigation into the IL-6 SNP rs1800796 variant reveals its association with EARR, suggesting a genetic influence on orthodontic outcomes, independent of the IL-1 pathway [28]. These genetic insights are complemented by hormonal considerations, such as the increased interleukin-1 activity in women post-ovulation, which may modulate the outcomes of orthodontic treatment due to estrous-cycle-dependent variations [29]. Such findings necessitate a personalized approach to orthodontic treatment planning, whereby genetic testing and consideration of hormonal cycles could optimize patient outcomes and mitigate the risk of EARR.

Polymorphisms within the IL-6 gene are associated with variations in inflammatory responses during orthodontic treatments, which in turn contribute to bone-resorbing effects potentially influencing the severity of EARR [4]. Moreover, these genetic variants do not act in isolation; they may interact with other genes or even within the same gene, further complicating the phenotypic outcomes related to root resorption [4]. Patients with certain IL-6 genotypes might have a heightened inflammatory response when subjected to orthodontic forces, leading to increased EARR, as observed in specific ethnic groups like the Chinese population [4].

The P2RX7 gene, which encodes a receptor involved in bone metabolism, is expressed in both osteoblasts and osteoclasts, the cells responsible for bone formation and resorption, respectively [1]. This expression pattern suggests that P2RX7 plays a crucial role in bone remodeling processes, which are inherently linked to dental health through the maintenance of tooth root structure. Moreover, P2RX7 appears to influence osteoblast function and promote osteoclast apoptosis, a dual action that could help maintain dental health by ensuring balanced bone remodeling [4]. The influence of P2RX7 genetic variants, specifically rs208294 and rs1718119, on EARR reveals a complex interplay of genetic predispositions across different populations. Notably, the GG genotype of rs1718119 has been associated with EARR in the Portuguese population, suggesting a potential genetic marker for susceptibility within this demographic [4]. Interestingly, while the CC and CT genotypes of rs208294 have shown a significant association with EARR in Caucasians, indicating a broader impact of this variant across various groups, it highlights the diversity in genetic influence within the P2RX7 gene [4]. The CG haplotype involving both SNPs rs208294 and rs1718119 further illustrates this complexity, as it shows a specific association with EARR in the Czech population [4]. Notably, the frequency of the C allele in P2RX7 rs3751143 did not differ significantly in patients who experienced particularly long treatment times, further indicating that this genetic factor does not correlate with extended orthodontic care [27]. Although the AA genotype of P2RX7 was more common among those with shorter treatment durations, this observation alone does not establish a definitive link between genotype and treatment length [27].

SNP rs1059703 in the X-linked IRAK1 gene emerged as a focal point, potentially affecting patients’ responses to orthodontic treatment and positively influencing the degree of root resorption observed [14].

The intricate interplay between RANKL, RANK, and OPG is central to understanding the pathophysiology of external apical root resorption, as it governs the balance between bone resorption and formation. RANKL, a key mediator, binds to the RANK receptor on osteoclast precursors, promoting their maturation into active osteoclasts, which are responsible for bone resorption [30]. OPG, a soluble decoy receptor produced by periodontal ligament cells, plays a critical role in regulating osteoclastogenesis by binding to RANKL, thus preventing it from interacting with RANK and subsequently downregulating osteoclast differentiation [31]. This regulatory mechanism is particularly significant in the context of periodontal diseases, where an imbalance in RANKL, RANK, and OPG levels can exacerbate pathological resorption of mineralized tissues, including the roots of teeth [30,31]. The communication facilitated by the RANKL/RANK/OPG system also extends to interactions with vascular and immune cells, highlighting its broader implications in dental pathophysiology [30].

A SNP for IL-1β, with a prevalence of around 9%, has been linked with EARR, underscoring its importance as a potential genetic marker [19]. Selected polymorphisms, including those from TNFRSF11B and IL-6, have been observed, with the C/T polymorphism for TNFRSF11B and the C/G polymorphism for IL-6 being the most prevalent [19]. Overall, while IL-1β emerges as a promising candidate for predicting EARR, the genetic contributions of TNFRSF11B, CASP1, and IL-6 require additional research to fully understand their involvement and to develop predictive models for EARR risk in orthodontic patients [19].

TNFSF11 and TNFRSF11B play a critical role in the OPG/RANKL/RANK signaling pathway. This pathway is essential in regulating osteoclast differentiation and maturation, processes that are pivotal for root resorption during orthodontic tooth movement [24]. For instance, the TNFSF11 rs12455775 polymorphism has shown a significant association with EARR [24]. Moreover, the TNFRSF11B rs2875845 polymorphism, although only nominally associated with EARR, aligns with previous findings, further indicating its relevance to root stability in orthodontic procedures [24].

The interplay between genetic factors and orthodontic procedures such as treatment duration and premolar extraction significantly influences the risk of OIEARR. Notably, the presence of premolar extraction has a complex interaction with treatment duration, where extraction status modifies the risk associated with the duration of treatment. For patients who undergo premolar extraction, the increased mechanical stress due to the larger distance canines and incisors must travel may heighten the risk of OIEARR; however, the duration of treatment in these cases plays a comparatively lesser role in influencing risk than in patients without extractions [21]. This complex relationship underscores the necessity of considering genetic profiles, particularly variants in genes such as OPG and RANK, which interact with treatment duration to affect OIEARR outcomes [21].

The investigation into the genetic underpinnings of EARR in orthodontic patients reveals noteworthy associations between specific vitamin D pathway gene variants and the degree of resorption experienced. The study underscores that individuals with the AA genotype in VDR rs2228570 exhibit a 21% higher incidence of EARR in lower first molars compared to those with AG and GG genotypes, suggesting a potential genetic predisposition to greater resorption [23]. In contrast, the presence of at least one G allele in CYP27B1 rs4646536 is associated with a 42% reduction in EARR of upper central incisors relative to the AA homozygotes, indicating that certain alleles may confer a protective effect against resorption [23]. These findings are significant despite not retaining statistical significance after adjustments for multiple testing. The main limitation of our study is that despite these findings, it is still not possible to use them for the establishment of new guidelines, and therefore there is no immediate practical hint for the clinician orthodontist.

## 5. Conclusions

Despite these advancements, it is still not feasible to establish new guidelines and clinical protocols based on the existing research findings. Future research should focus on longitudinal studies that not only elucidate the genetic underpinnings of EARR but also explore the interplay of environmental factors and treatment variables over time, thus enabling us to integrate genetic considerations into orthodontic practice. Ultimately, the integration of genetic considerations into orthodontic practice has the potential to revolutionize treatment strategies, ensuring that they are not only effective but also respectful of each patient’s unique biological landscape.

## Figures and Tables

**Table 1:** Studies included in the comprehensive review.

430 articles through keywords	
Time frame 2014–2024	
274 articles remained	156 articles excluded
The rest of inclusion criteria	
16 articles remained	258 articles excluded

The features of the included articles were summarized in Table 2.

**Table 2:** Summary of the studies included in the comprehensive review.

Study	Country	Year	Location of Study	Type of Study	Cases	Controls	Gene Polymorphism	Conclusion
Iglesias Linares et al. [11]	Spain	2014	University clinic	Case control	37	50	SPP1 (rs9138, rs11730582)	SPP1 (rs9138, rs11730582) are correlated to EARR
Sharab et al. [12]	USA	2015	Private orthodontic practice	Case control	67	67	P2RX7 (rs208294, rs1718119, rs2230912); CASP1/ICE (rs580253, rs530537); CASP5 (rs554344); IL1B (rs1143634); IL1A (rs1800587); IL1Ra (rs419598)	P2RX7 rs208294 is correlated to EARR
Guo et al. [13]	China	2016	University clinic	Case control	174		IL1RN (rs419598); IL-6 SNP (rs1800796)	IL-6 SNP rs1800796 is correlated to EARR
Pereira et al. [14]	Portugal	2016	University clinic and private orthodontic practice	Cross sectional	195		IL1B (rs1143634); IL1RN (rs315952); IRAK1 (rs1059703)	IRAK1 Polymorphism may protect against EARR
Iglesias Linares et al. [15]	Spain	2017	University clinic and private orthodontic practice	Case control	174	198	IL1B (rs1143634); IL1RN (rs419598); SPP1 (rs9138/rs11730582)	Homozygous IL1RN (rs419598) manifested more commonly EARR
Borilova Linhartovaet al. [16]	Czech Republic	2017	University clinic	Case control	30	69	IL-17 (rs2275913); SPP1 (rs11730582, rs9138); P2RX7 (rs208294, Tyr155His, rs1718119); 11B (rs3102735, rs2073618)	P2RX7 may contribute to EARR
Borges de Castilhos et al. [17]	Brazil	2019	University clinic and private orthodontic practice	Case control	178	160	RANKL; RANK; OPG	RANK and OPG were correlated to EARR
Iber-Diaz et al. [18]	Spain	2020	University clinic	Cohort	101	361	STAG2 (rs151184635); RP1-30E17.2 (rs55839915)	Genes on chromosomes X and Y may induce aggressive EARR
Ciurla et al. [19]	Poland	2021	Private orthodontic practice	Case control	101		IL-1β; TNFRSF11B; CASP1; IL-6	IL-1β gene is correlated to EARR
Ciurla et al. [20]	Poland	2021	Private orthodontic practice	Case control	40	61	IL1RN (rs419598); P2RX7 (rs208294)	P2RX7 and IL1RN are correlated to EARR
Silva et al. [21]	Portugal	2022	University clinic and private orthodontic practice	Cross sectional	195		IL1B (rs1143634); OPG (rs3102735); IL1RN (rs315952); RANK (rs1805034); P2RX7(rs1718119)	Genetic predisposition is influenced by clinical variables
Yun-Ju Lee et al. [22]	Korea	2022	University clinic	Cohort	118		34 genes and their polymorphisms	SPP1 rs9138 and SFRP2 rs3810765 are correlated to EARR. Genes regulating inflammation and the Wnt signaling affect the degree of EARR.
Maranon Vasquez et al. [23]	Germany	2023	University clinic and private orthodontic practice	Cross sectional	143		VDR (rs731236), (rs7975232), (rs1544410), (rs2228570); GC (rs4588); CYP27B1 (rs4646536); CYP24A1 (rs927650)	No statistical significance after multiple testing
Liu Jing et al. [24]	Korea	2024	University clinic	Cohort	77		TNFSF11 (rs2296533), (rs3742257); TNFRSF11B (rs2875845); WNT3A (rs4653533); SFRP2 (rs3810765); P2RX7 (rs17525809), (rs208294), (rs1718119); LRP1 (rs1800137), (rs1800141); LRP6 (rs2302685)	NFSF11, TNFRSF11B, WNT3A, SFRP2, LRP6, P2RX7, and LRP1 are correlated to EARR
Jyoti Chauhan et al. [25]	India	2024	University clinic	Cohort	142		IL-1β (rs 1143634); IL-1α (rs1800587)	No correlation was found
John M Burnheimer et al. [26]	USA	2024		Cohort	218		Several SNPs	ACT3N rs678397 and TSC2 rs1051771 are correlated to EARR
